# Damage processes in extended laser exposures using an *in vitro* model

**DOI:** 10.3389/fopht.2025.1435692

**Published:** 2025-08-08

**Authors:** Nathaniel J. Pope, Jin Ha, Madeline E. Melzer, Priscilla Lopez, Amanda Tijerina, Gary D. Noojin, Michael L. Denton

**Affiliations:** ^1^ Biosciences Department, Science Applications International Corporation, JBSA-Fort Sam Houston, TX, United States; ^2^ Rosenberg School of Optometry, University of Incarnate Word, San Antonio, TX, United States; ^3^ Department of Cell and Developmental Biology, Feinberg School of Medicine, Center for Synthetic Biology, Northwestern University, Chicago, IL, United States; ^4^ Department of Biomedical Engineering and Chemical Engineering, University of Texas at San Antonio, San Antonio, TX, United States; ^5^ BioResearch, Conceptual MindWorks, Inc., San Antonio, TX, United States; ^6^ Bioeffects Division, Air Force Research Lab, JBSA-Fort Sam Houston, TX, United States

**Keywords:** laser, RPE cell damage, photothermal, photochemical, irradiance reciprocity, concurrent exposures, Probit threshold, microthermography

## Abstract

Retinal pigment epithelial (RPE) cells are sensitive to both photothermal and photochemical damage when exposed to lasers with wavelengths associated with the retinal blue light hazard. Laser power density (irradiance) and exposure duration primarily dictate the damage mechanism. Relatively high irradiances and short exposure durations typically lead to melanin-dependent photothermal damage, whereas low irradiance and long duration exposures are required for photochemical pathways. However, little is known about damage mechanisms at intermediate irradiances and durations for pigmented cells. The current Z136.1–2022 laser safety standard from the American National Standards Institute (ANSI) does not consider combined photothermal and photochemical damage processes. In addition, the ANSI Z136.1 standard classifies photochemical damage as nonthermal. Here, we use extended laser exposure parameters in an *in vitro* RPE cell model (ATCC CRL-4000) to show that elevated temperatures accelerate photochemical damage mechanisms. In addition, for 447-nm exposure conditions leading to damage considered neither purely photothermal nor photochemical, there is a reduced requirement for the thermal component for cell death. Our results suggest the need to address safety for lasers with blue wavelength emission, as in ophthalmic devices.

## 1 Introduction

The retina is sensitive to laser damage (1, 2), especially the pigmented cells of the retinal pigment epithelium (RPE). For exposures longer than 10 µs this damage generally occurs via either photothermal or photochemical mechanisms (1). Photothermal damage results from laser-induced heating, typically due to absorption by melanin or bulk water depending on the wavelength of the laser in use (2, 3). When the tissue temperature exceeds certain thresholds cell death can occur (4, 5). This process is largely governed by the combination of irradiance and exposure duration, with high irradiances over short periods being most effective. In contrast, photochemical damage is initiated by absorption of high-energy photons that excite cellular chromophores, generating reactive oxygen or nitrogen species (RXS) or causing direct oxidative modification of cellular structures (6, 7). This process typically dominates under low irradiance and long-duration exposures and does not require bulk tissue heating.

Photothermal damage can be induced via absorption of light by intracellular melanosomes or bulk water across the visible (VIS) to near infrared (NIR) spectrum, which produces elevated temperatures in the RPE (1, 2). It has been hypothesized that the molecular mechanism underlying photothermal damage is due to heat-induced unfolding and aggregation (denaturation) of critical cellular proteins, with concomitant loss of function (3–5), however, no specific protein targets have been identified.

In contrast, photochemical damage requires short wavelength light, generally in the blue to ultraviolet range. Specific photoexcitation reactions proposed to generate oxidative products are diverse and, due to their requirement for high photon energy, are wavelength dependent. All cells are sensitive to photochemical damage processes by short wavelength VIS light (6, 7), but the presence of melanosomes may also elicit additional photooxidative stresses (1, 8–10). Though generally considered protective, the melanin pigments within the melanosomes can act as a photosensitizer under some conditions (1, 8–10). Whether due to production of RXS or direct oxidation of protein side chains, a likely damage outcome is protein inactivation via oxidation (7). This spectral dependence for photochemical damage at the retina defines the blue light hazard (11).

Typically, laser photochemical damage is considered a non-thermal process (12), but the melanosomes of the RPE make blue light a double threat for photochemical and photothermal laser damage (8–10). A common metric for estimating laser photothermal damage is a 10 °C rise in temperature above ambient (2). Others have proposed that purely photothermal damage requires a 20 °C temperature rise, while the range of 10-20 °C may generate both photothermal and photochemical mechanisms (13). Supporting this notion, using confocal microscopy we have previously demonstrated that cultured RPE cells continue to exhibit photooxidation at ambient temperatures up to 50 °C, with some differences based on pigmentation (14). In that study, the maximum ambient temperature at which photooxidation persisted was not determined due to experimental constraints.

Currently, the ANSI Z136.1 laser safety standard (15) does not consider the potential for concurrent photothermal and photochemical damage. Calculation of maximum permissible exposures (MPEs) provide a level of safety because they are set well below known hazardous levels. The ANSI Z136.1 standard recognizes that simultaneous exposure to pulses and continuous wave (CW) lasers can act synergistically. However, the methodology currently prescribed for photothermal and photochemical damage uses a dual limits system. This means that the MPEs for photothermal and photochemical processes are calculated independently, and the most restrictive one is implemented for safety. We are interested in determining whether both processes contribute to damage for laser exposures in the blue spectrum. To do this, we used a simple *in vitro* model of laser eye injury with extended laser exposure conditions that are not representative of what can be achieved in native retina.

Although little is known about whether photothermal and photochemical processes can concurrently contribute to cellular damage, a sharp transition from photothermal to photochemical laser damage has been reported for various experimental models (13–17). Our recent advancement (18) of a prior computational model for this transition is available as a companion paper in this issue (19). Our *in vitro* laser damage model is comprised of artificially pigmented RPE cell cultures, specifically hTERT RPE-1 (ATCC CRL-4000). Previous studies have demonstrated that this model approximates damage trends seen in animal models and is useful in studying laser damage because it allows rapid assessment of laser-dose responses (20, 21). Unfortunately, the *in vitro* model cannot simulate the full range of physiological and humoral responses of the retina. However, the model does permit analyses of RPE cell laser sensitivities with flexible and accurate laser delivery and dosimetry, with cell imaging modalities such as fluorescence detection of damage (20, 21) and thermography (22). The *in vitro* approach has provided a means of determining threshold temperatures leading to photothermal damage (23, 24), which we utilize in the current study.

Computational models are an important adjunct to empirical studies. Modeling temperature-dependent damage using the Arrhenius second-order rate constant has long been used in laser damage studies. The damage integral is a measure of thermal damage accumulation and has been described in detail elsewhere (23). A recent paper (19) adopts the damage integral model for both photothermal and photochemical damage for simulated thermal responses. This combined damage integral is the first known report for using the damage integral to represent photochemical damage processes. Although the model requires further validation, the method assumes that inactivation of important cellular proteins is the mechanism behind both photothermal and photochemical damage. An important outcome of the combined damage integral is the use of photon flux (number of photons delivered per second) as a mathematical switch, indicating if an exposure is purely photochemical in nature.

In this paper, we use a variety of *in vitro* methods to determine laser threshold dose and temperature to study the interplay between photothermal and photochemical mechanisms of cell damage. Finally, using simultaneous exposure to lasers at both 2 μm and 447 nm, we tested if the combination of photothermal and photochemical processes could produce synergistic or additive damage outcomes. Our results will increase the understanding of laser damage mechanisms for groups such as the ANSI Z136.1 subcommittees evaluating safe laser practices in humans, and clinicians wanting to diagnose potential sources of retinal laser lesions.

## 2 Materials and methods

### 2.1 Exposure apparatus

**Figure 1:**
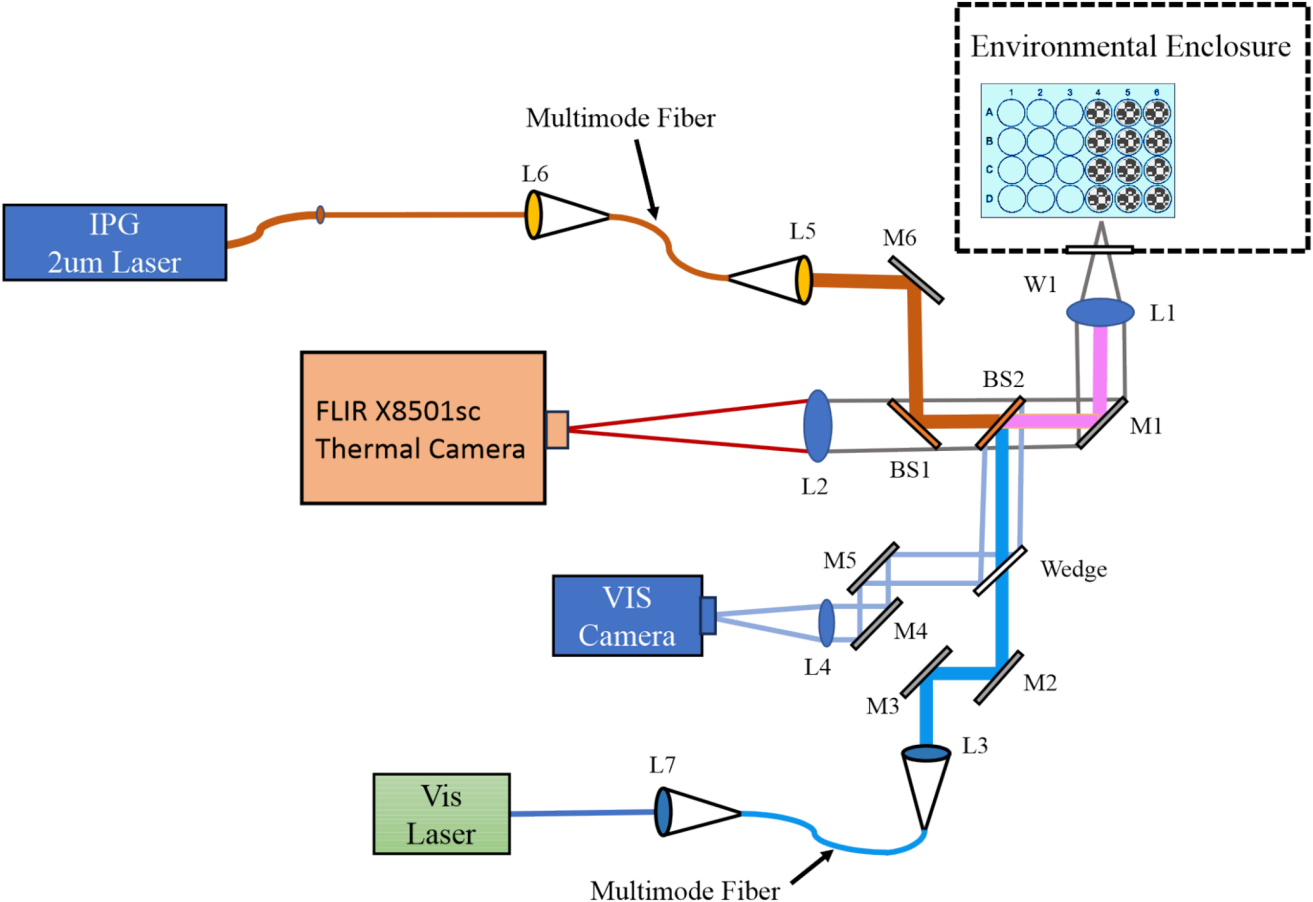
Dual wavelength laser delivery system. The in-house developed microscope delivered both a NIR (2 µm) and a VIS (447 nm) laser beam. Both laser systems were imaged to the same focal plane and region of cultured cells via imaging of the output of multimode fibers. Imaging modalities included a thermal camera (FLIR) running at 1,000 fps in real-time with laser exposures, and a visible camera for focus and beam alignment. L; lens, M; mirror, BS; beam splitter, W; optical window.

Laser delivery and cell imaging were combined in a four-armed microscope system by essentially merging the two different experimental systems presented previously (23). **Figure 1** represents the exposure apparatus, which included a large Plexiglass environmental enclosure used for exposures (drawn as a dashed line) that provided consistent ambient temperatures around 35 °C and 70% relative humidity as previously described in detail (25). Also within the enclosure, a sample holder fitting any standard size microtiter multiwell plate, shown as a blue plate with 24 circles (rotated 90°for effect), was mounted on a computer controlled 2-axis (XY) translational stage. The stage reproducibly moved our glass bottomed 24-well plates (Cellvis, P24-0-N) enabling laser delivery and imaging of cells in the center of each well. The interface between the interior (cells) and exterior (cameras and lasers) of the enclosure was an optical window (W1) made of calcium fluoride. A third axis of the stage was a z-micrometer allowing fine focus of cells that ensured proper co-focus of lasers and cameras. This also ensured the laser profile and beam diameter was consistent at the cellular image plane.

The microscope arms allowed for concurrent delivery of lasers with wavelengths in the VIS and NIR spectrum. For VIS exposures in the current work, we used either a 447 nm (Opto Engine LLC, MDL-F-447-2W) or a 647 nm (Coherent Innova Technology Saber, SBRC-PL) laser launched into a multimode 400-µm core diameter (Thorlabs Inc, FG400UEP) or 200-µm core diameter (Thorlabs Inc, FG200UEP) High OH fiber-optic cable, each with numerical aperture of 0.22. The output of the fiber was relay imaged to the co-focal sample plane in the environmental enclosure producing a flat-top beam profile. Lenses L1 and L3 imaged the fiber tip to the cellular plane, which were in an infinity configuration. The resulting images using the 400-µm core diameter fiber were 825 ± 14 µm in diameter at the sample plane. Resulting images using the 200-µm core diameter fiber were 416 ± 8 µm (447 nm) and 415 ± 21 µm (647 nm). A shutter before the VIS laser fiber launch controlled the exposure duration via computer control. Reference meter 1, with its associated beam splitter, was placed before the shutter in the VIS laser path. This allowed setting the desired exposure irradiance using a ratio between reference meter 1 and the sample position meter. This was measured each exposure day the VIS lasers were used. The reference meter also monitored the laser power during the exposures, and exposure metadata were written to a file after the exposure.

The NIR laser arm was optimized to deliver a NIR wavelength laser to the sample plane at the same position as the VIS laser delivery arm. The current study used a 20 W 2-µm laser (IPG Photonics, TLR-20-2000-LP). The 2-µm laser was launched into a Low OH 400-µm core diameter multimode 0.22 NA fiber-optic cable (Thorlabs Inc, FG400LEP) by L6. The output of this fiber was relay imaged by L5 and L1 in the same manner as the VIS lasers. The relay imaging produced a 726 ± 13 µm diameter flat-top beam profile at the sample plane. A computer-controlled shutter inserted between L6 and the laser’s output controlled the exposure duration. Laser irradiance was monitored during laser exposures with beam splitter BS3 and reference meter 2, and exposure metadata were written to a file after the exposure. The laser irradiance was set with the 2-μm laser’s controller. Ratios between the reference meter 2 and the sample position meter were measured each day before using the 2-μm laser.

The differences in the diameters between the 2-µm (726 ± 13 µm) and the large 447-nm (825 ± 14 µm) beams stem from the imaging modalities for their delivery. The laser delivery optics used were chosen to ensure the output of the fiber-optic was imaged to the same focal plane as the video and thermal cameras while generating a flat top image profile with uniform power density. As such, the only way to change the laser spot size was to change the core diameter of the visible or NIR fiber-optic and select the correct ratio of available imaging optics for the laser wavelengths in use. The major limiting optic was the final objective of the microscope, which had a fixed wavelength-dependent focal length. This meant that the step sizes for the beam diameters were coarse. We chose to have the 447-nm beam to be the larger of the two so that the thermal responses would not exceed the photochemical boundaries. On average, the 2-µm beam was only 12% smaller than the 447-nm beam.

For reporting accurate laser irradiances, both beam radius and laser power at the sample must be measured routinely. Beam radii were regularly measured in the x and y dimensions using the knife-edge method (26), taking advantage of the XY translational stage and a custom LabVIEW program. Measurements were repeated three times in each dimension, and these values were averaged to calculate the final dimensions of the laser beam. Prior to each day of exposures, a power calibration curve was prepared by measuring and comparing power at a reference detector and at the sample plane (without the microtiter plate). A broad range of powers were measured at the reference and sample detectors and the ratio, along with the laser radius, was used to calculate the laser irradiance in real time (LabVIEW). The overall uncertainties (27) associated with determining laser irradiance for all the laser wavelengths and diameters were 2.8-3.2%. When including the uncertainties of the laser shutter device, the uncertainties (27) for determining laser radiant exposure were 3.5-3.8%.

The mid-wave (3-5 µm) thermal camera (Teledyne FLIR LLC, X8501sc) imaged temperature distributions at the sample plane with a frame rate of 1,000 frames per second and an effective pixel pitch of 6x6 µm at the sample plane (L1, L2, BS1, BS2, M1, W1). With this magnification, temperatures were identified spatially and temporally (23). The thermal camera was calibrated as previously described in detail (22). Additionally, the uniformity of the laser profile was assessed by microthermography using a piece of copper foil painted with flat black paint.

A VIS light imaging arm (L1, M1, BS2, Wedge, L4, M4, M5) operated parallel to the visible laser delivery arm, providing bright field microscopy of the sample at up to 60 frames per second. Images were captured in real time prior to, during, and after laser exposures. Both laser arms and the thermal camera were focused to the same plane as the VIS camera. This allowed confidence in delivering previously measured beam diameters and accurate thermal data when focusing on cells using the VIS camera.

### 2.2 Cell culture and laser exposures

#### 2.2.1 Maintaining cultures

All exposures used a previously published *in vitro* model of laser retinal damage (20, 21) featuring artificially pigmented hTERT-RPE1 cells (ATCC, CRL4000). hTERT-RPE1 adherent cells were cultured in flasks and plates in DMEM/F12 50/50 base medium without L-glutamine (Corning, 15-090-CM). To make complete medium, we added 10% fetal bovine serum (R&D Systems, S11150) and supplemented to final concentrations of 10-mM HEPES buffer (Fisher, BP-299-100), 100-μg/ml (each) penicillin/streptomycin (Corning 30-002-CI), and 50-μg/ml gentamycin (Corning, 30-005-CR). Supplementation for L-glutamine was carried out by adding GlutaMAX (ThermoFisher, 35050061) to the manufacturer’s suggested concentration. All manipulations were carried out in a standard biological safety cabinet (Labconco Logic Class II Type A2) with incubation in a standard air-jacketed cell culture incubator (Thermo Scientific HERAcell 150i).

Cells were split at a ratio of 1:20 every 3–4 d (80% to 90% confluency). Cells were grown at 37°C in a humidified incubator (Thermo Scientific HERAcell 150i) with 5% CO_2_. To split cells, medium was removed via aspiration and cells were rinsed with sterile Dulbecco’s phosphate-buffered saline (DPBS, Corning, 21-030-CM), followed by aspiration. Addition of 0.05% Trypsin EDTA (Corning, 25-052-CV) was followed by incubations for 5–10 min at 37°C. Complete medium (four times the volume of trypsin) was added to stop the trypsinization process. Cells were dispersed into single-cell suspensions by triturating with a pipette and then inoculated into new flasks/dishes as needed. Cells for exposures were only used between passage number 15-31.

#### 2.2.2 Preparation for exposure

Cells for exposures were plated into #0 thickness glass bottom 24 well plates (Cellvis, P24-0-N) two days prior to exposure. Before seeding sample plates, a small aliquot of the cell suspension was diluted and cells were counted using a Beckman Coulter, Z1 Coulter Particle Counter (5 replicates per sample). The cell suspension stock was adjusted to 100,000 cells per mL in complete medium and 1 mL was seeded into the wells of plates. This process marked “day 0” relative to subsequent steps before laser exposure. On day 1 post seeding, cells were artificially pigmented (phagocytosis) with melanosome particles (MPs) isolated from bovine eyes as previously described (8), which mimic endogenous melanosomes, and laser exposures occurred on day 2. The well designated A1 was always left empty to allow spot calibration (digital offset) of the thermal camera before each exposure, which has been previously described in depth (22, 25). Based on the experimental design, the number of plates and wells to be seeded on day 0 was limited such that cells would not be out of the incubator for more than 60–90 min on exposure day.

On day 1 post seeding, cells in all wells were visually inspected for general health. Wells with healthy cells were artificially pigmented with isolated MPs, as described previously (8, 17). Cells were allowed to phagocytose MPs overnight. Prior studies showed that MP localization changes upon phagocytosis, and MPs change from a random even distribution on the cell surface to specifically localized in the perinuclear region of the cytoplasm (8, 17, 20). The number of MPs added per well is based both on the expected number of cells in each well the day of the laser exposure (3-4x10^5^ cells on day 2 after continued growth), and the concentration of each MP stock solution (typically 9x10^9^ MP/mL). The final number of MPs added was about 165 ± 35 MPs per cell (5.6x10^7^ MPs per well). Due to varying cell number per well and errors in counting stock solutions of 1-µm size MPs, damage sensitivity (see below) for each new batch is compared with that of the previous batch to determine similar bulk absorption properties in the RPE cells. In this way, 165 MPs per cell relates directly to the 250 MPs per cell in a prior publication (28).

On day 2 post seeding, cells in all wells were visually inspected for general health. Cell morphology was also observed, and perinuclear MP location was confirmed, to ensure MPs were phagocytosed. Immediately prior to exposure, medium was removed by aspiration and cells were washed three times using 0.5 mL of exposure buffer to remove any residual MPs in the solution or loosely deposited on the cell surface. Exposure buffer consisted of Hank’s Balanced Salt Solution (HBSS) with calcium and magnesium, without phenol red or sodium bicarbonate (Corning, 20-023-CV). The buffer was diluted using 18.2 MΩ·cm water and filter sterilized. The first two washes were performed in the biological safety cabinet using prewarmed (37°C) buffer. The last wash occurred in a portable hood near the exposure enclosure using prewarmed buffer. The plates were then placed in the exposure box to thermally equilibrate for at least ten minutes prior to exposure. Progression of thermal equilibrium could be assessed using the thermal camera focused on cells.

After laser exposures, plates were removed from the exposure box and the exposure buffer was replaced with prewarmed complete medium in the biological safety cabinet. Cells were allowed to respond to injury in the incubator for 60–80 min.

#### 2.2.3 Laser exposure

For concurrent exposures at 447 nm and 2 µm, the beam diameters were 825 ± 14 µm and 726 ± 13 µm, respectively. For identifying irradiance reciprocity for 447 nm at 200 s and 400 s the same beam was used as in the concurrent exposures (825 ± 14 µm). For threshold peak temperature determinations at 447 nm and 647 nm, laser beam diameters were 416 ± 8 µm and 415 ± 21 µm, respectively.

### 2.3 Damage assessment

#### 2.3.1 Cell staining and microscopy

Cells were stained with a combination of standard live and dead viability stains. Calcein-AM (Invitrogen, C3100MP) indicated live cells (green cytoplasm) and ethidium homodimer-2 (Invitrogen E3599) indicated dead cells (red nuclei). Ethidium homodimer-2 was purchased as a ready to use 1 mM stock in dimethyl sulfoxide (DMSO). To prepare a stock solution of calcein-AM for use, 50 µl of DMSO (Sigma, D2650-5X5ML) was added to a 50-µg vial of calcein-AM and vortexed to resuspend. Calcein stock solution was briefly centrifuged to collect the full volume at the bottom of the tube. Calcein is stable at -20 °C in the lyophilized form, but once suspended in DMSO for use, it was used within 10 days.

Immediately before staining cells, 0.73 µl of the calcein stock solution, and 1.4 µl of the ethidium homodimer stock was added per 1 mL of room temperature exposure buffer. Cell culture medium was removed by aspiration and replaced with 0.5 mL per well exposure buffer with added dye solutions. Plates were returned to the incubator for 20 min. After the incubation, plates were either washed and kept at room temperature until they could be imaged or imaged immediately.

Viability was assessed using an Olympus UPlanFLN 4x objective on an Olympus IX-73 inverted fluorescence microscope using FITC (calcein-AM) and Cy5 (ethidium homodimer) filters. Unexposed wells were used as a negative control and had low (< 1%) rate of spontaneous cell death. For consistency, images were acquired with excitation lamp intensity set to 50% with an exposure duration of 300 ms. Wells were scored for presence or absence of an *in vitro* “lesion” using a consensus of two scorers. A binary score of laser irradiance and yes or no damage was used in Probit analysis (see below).

#### 2.3.2 Probit analysis and lesion scoring criteria

The Probit method is a probabilistic method to analyze binary data (irradiance versus yes/no damage) (29, 30). The Probit estimated doses of ED_50_ and ED_25_ provide laser (irradiance) doses expected to produce damage 50% and 25% of the time, respectively, when using the same experimental parameters used to generate the Probit input data. Other important Probit parameters are the slope (first derivative) of the probability curve at 50% and the upper and lower 95% confidence intervals (fiducial limits). The damage threshold determinations were considered final when the slope was greater than five and the relative fiducial limits were within ±30% of EDxx values of interest.

Since the lesions were scored on a binary basis, the criteria for scoring needed to be clear and well defined. In this study, photothermal damage appeared straightforward. Due to thermal diffusion geometry, the highest temperatures were reached in the center of the exposed site in all exposures. As irradiance was increased thermal damage appeared as cells died (red stain) initially in the most central regions and expanded radially. Some sublethal outcomes (calcein staining of contracted cells) were observed surrounding the centralized dead cells. However, if this morphologic alteration was not accompanied by overt damage (red ethidium stain) centrally, the outcome was considered negative.

Our data show photochemical *in vitro* lesions with substantially different morphology and were scored by different criteria. At maximum damage outcomes, morphology consisted of contiguous dead cells covering most of the area exposed by the laser, but without live cells having altered morphology surrounding the dead core seen with photothermal damage. As laser irradiance was reduced, such as near and below the Probit ED_50_ value, dead cells were often found scattered randomly throughout, again without the halo of altered live cells seen with photothermal responses. Only outcomes from the 447-nm exposures exhibited this morphology and were therefore scored as positive for damage. Minimally damaged *in vitro* lesions were scored positive for photochemical damage if the effected region had an identifiable circular shape (like the laser), if the size of the circular damage did not exceed the dimensions of the laser footprint, and if it contained at least 5 dead cells intermixed within the center of that region.

For both photothermal and photochemical *in vitro* lesions, wells were discarded from consideration if they had obvious defects preventing clear scoring. For example, wells were excluded if there were disruptions to the cell monolayer, clumps of MPs in or near the laser footprint, foreign material in or near the laser footprint, or signs the staining or washing processes had caused mechanical disruption of the cell monolayer.

#### 2.3.3 Damage frequency

To assess changes in the laser sensitivity of the *in vitro* model without determining new Probit ED_xx_ values we utilized the reliability of the Probit threshold process. To ensure this functionality, we assessed damage frequency with ED_50_ and ED_25_ irradiances to confirm they generated approximately 50% and 25% damage outcomes, respectively. Then we used the damage frequency method in conjunction with the combined 2-µm and 477-nm exposures to identify changes in damage sensitivity and assess for concurrent damage processes.

### 2.4 Thermal data assessment

Using methods previously established (23, 24, 28), we determined temperatures at the boundary of cell death using overlays of real-time microthermography movies and fluorescence images identifying contiguous dead cells postexposure. These damage threshold temperatures are taken from a single-pixel region of interest (ROI) from the thermal movies that corresponded to the outer boundary of the damaged areas.

Recent advances in the postexposure image processing removed much of the subjectivity involved in determining threshold temperature metrics. In brief, this was accomplished using a custom ImageJ (31) script. To construct the damage masks the script first split the fluorescence channels, then subtracted the red channel from the green channel to eliminate any bleed-through. The contrast was then enhanced, and four threshold maps were computed using the RATS (Robust Automatic Threshold Selection) algorithm included in ImageJ. These were set up to use slightly more strict parameters with each iteration, such that the first mask generated had the most edge feature detail and fidelity. Each subsequent mask had slightly less stringency and thus a larger minimum feature size. Each of these masks was then post-processed by running a “cleaning” algorithm that would run a series of 10 erode operations (removing the outermost pixels of the binary mask), then 10 dilate operations (add additional pixels around the edge of the binary mask). This served to remove any random noise pixels or stray projections from the mask, while not changing the overall size of the damaged region. Finally, visual inspection ensured only one contiguous area per mask remained, and any voids within that area were filled. If this automated process could not generate a single contiguous mask area, or included obviously spurious adject regions, these were filled or removed by hand.

The four damage masks were then superimposed on the thermal video file output using a custom LabVIEW program. This program first identified the hottest (central) point of the thermogram for a given exposure. It then rotated, flipped, and scaled the mask to have the same orientation and (resolution) scale as the fluorescence damage image for the same exposure. The software then aligned the thermogram with each of the four damage masks previously generated. The mask with the smallest standard deviation for temperatures within the thermal boundary ROI was chosen for temporal batch extraction from corresponding thermal movies later.

## 3 Results and discussion

RPE cells can respond to blue wavelength laser light by generating heat due to absorption by MPs or by generating oxidative reactions on proteins directly or indirectly from small RXS molecules. Common dogma suggests that laser irradiance and exposure duration play a causal role in dictating blue light damage. High irradiances that lead to significant temperature rise lead to damage because the irreversible denaturation reactions occur rapidly and inactivate proteins. Photochemical damage is often considered a nonthermal process. Therefore, low irradiances for long exposure times allow accumulation of photochemical products with little or no temperature rise. Little is known about if, or how, the two damage processes contribute to death pathways when temperature rise is significant but not severe, and exposures are long enough to accumulate substantial levels of photochemical products. We have devised an experiment to address whether combining the different damage pathways influences damage outcomes. This required clearly defined endpoints that distinguish purely photothermal from purely photochemical damage, and a method to expose cells to two lasers concurrently. We have accomplished this by using long exposures (200 s) to 447 nm and 2 µm laser light simultaneously.

Using Probit ED_50_ damage thresholds at 447 nm we identified irradiance reciprocity between exposures at 200 s and 400 s, indicating purely photochemical damage. The principle of irradiance reciprocity, originally described as the Bunsen-Roscoe reciprocity law (32), and subsequently adopted by Ham (13) and Blankenstein (33) for laser exposures, basically states that a purely photochemical process is a function of the number of photons delivered. As summarized by Blankenstein, “for an exposure duration of 2t, the ED_50_ will be 0.5 E_0_,” where t is exposure duration and E_0_ is laser irradiance at t. There are known limitations to this principle but when irradiance reciprocity is found, this is a strong indication that a given process has a photochemical mechanism. We have previously reported irradiance reciprocity in laser damage thresholds (17). In essence, when irradiance (W cm^-2^ = J s^-1^ cm^-2^) is multiplied by the exposure duration the radiant exposure (J cm^-2^) is given. Radiant exposure can be a measure of the number of photons delivered if comparisons are made within the same wavelength because the photon energy is the same. Reciprocity occurs when two damage threshold radiant exposure values are the same.

Our use of laser exposures at 2 µm generated purely photothermal damage due to bulk water heating (vibrations) and low photon energy relative to blue wavelengths. Measuring temperatures in real-time with laser exposure (microthermography) provided thermal histories of each exposure, which allowed the determination of threshold temperatures. Finally, we have constructed a laser delivery system allowing concurrent exposures at 447 nm and 2 µm (**Figure 1**).

### 3.1 Cell damage

**Figure 2:**
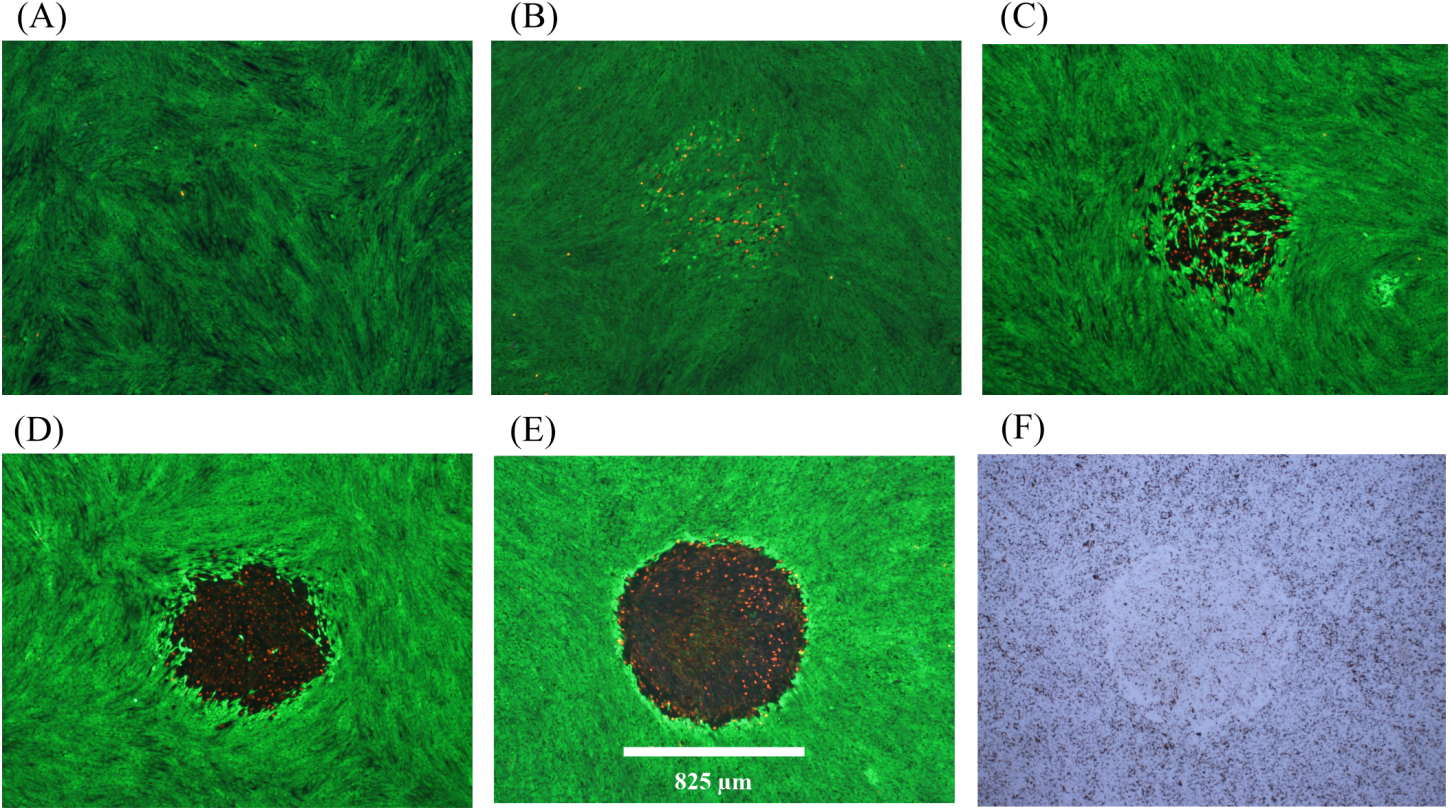
Damage assessment for 200-s exposures to 447 nm (825-µm diameter). **(A)**: Not exposed. **(B)**: 8.84 W cm^-2^ (Probit ED_25_), damaged. **(C)**: 10 W cm^-2^ (Probit ED_50_), damaged, **(D)**: 14 W cm^-2^, damaged. **(E)**: 15 W cm^-2^, damaged. **(F)**: Bright-field image for Panel **(E)** Spatial bar: 825 µm (beam diameter).

**Figure 3:**
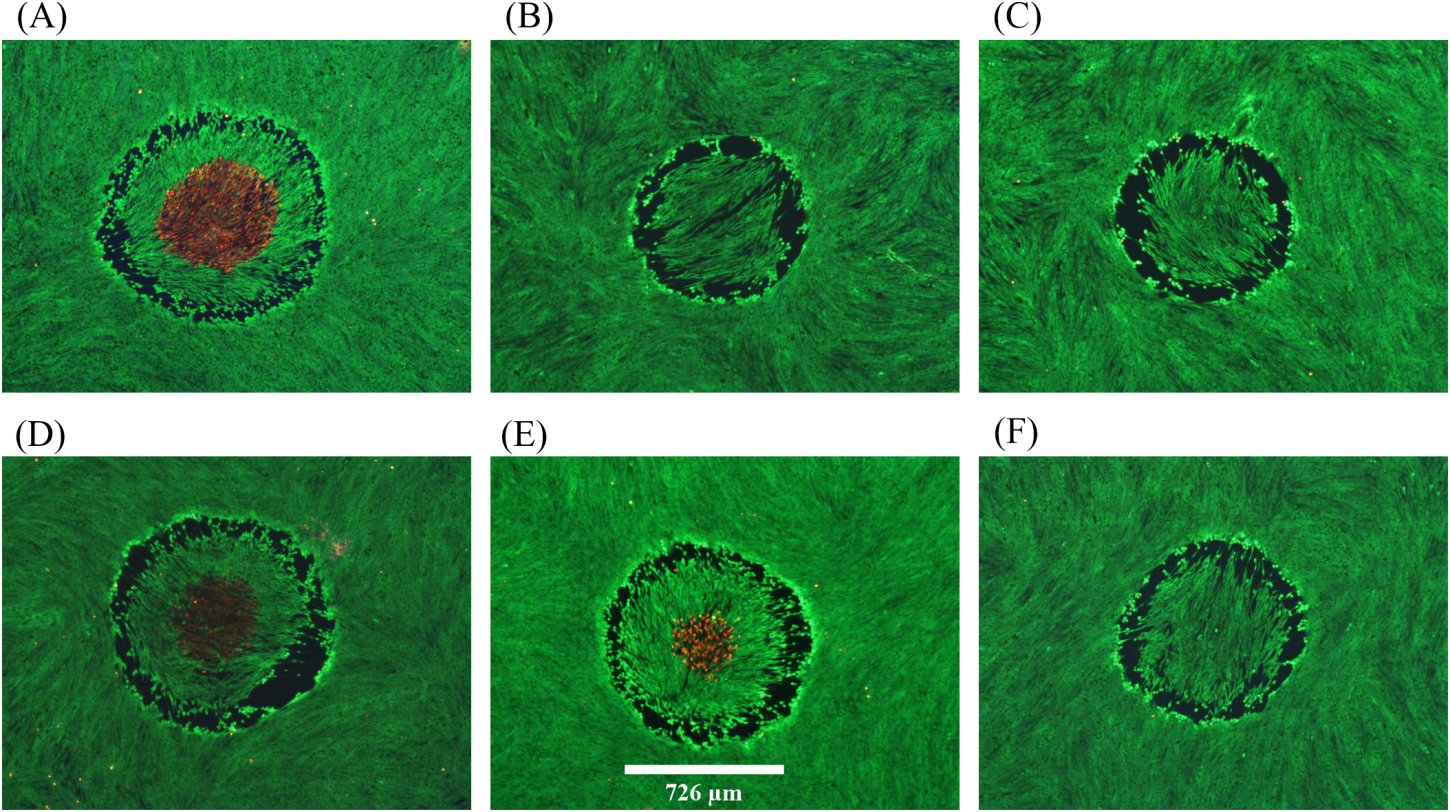
Damage assessments at near threshold irradiance for 200-s exposures at 2 µm. **(A)**: 12.15 W cm^-2^, damaged, 51.0 °C. **(B)**: 12.27 W cm^-2^, no damage. **(C)**: 12.27 W cm^-2^, no damage. **(D)**: 12.27 W cm^-2^, damaged, 49.4 °C. **(E)**: 12.27 W cm^-2^, damaged, 49.6 °C. **(F)**: 12.29 W cm^-2^, no damage. Red indicates dead cells and green indicates live cells. Spatial bar: 726 µm (beam diameter).

Descriptions of damage morphologies used for scoring damage were provided in Section 3.3.2. **Figures 2** and **3** provide examples of *in vitro* laser damage for purely photochemical (200-s exposure at 447 nm) and purely photothermal (200-s exposure at 2-µm) processes, respectively. Viability staining of unexposed cell monolayer is represented in **Figure 2A**. With increasing laser irradiance, the progression from minimal (**Figure 2B**) to maximal (**Figure 2E**) photochemical damage is illustrated. **Figure 2F** shows the bright-field image corresponding to the exposure shown in panel 2E. Notice that in minimal photochemical damage (**Figure 2B**), numerous dead cells (red) were intermixed amongst living cells (green), and the slight hyperfluorescent surviving cells clearly demarked the laser exposure site. Maximal photochemical damage has contiguous dead cells in a region about the same area as the 447-nm laser beam (**Figure 2E**) and produces significant alterations in bright-field morphology (**Figure 2F**). These results are similar to previous images of photochemical damage in the *in vitro* model of laser retinal damage (20, 21, 33), although our current assessments are more thorough and use glass-bottom plates rather than plastic.

There were 447-nm exposures that produced the hyperfluorescence of cells in the center region that was not accompanied by dead cells. We chose to require a significant number of dead cells to consider a lethal event for these overt (1-h postexposure) damage assessments. Staining for subtle damage, such as apoptosis is complicated by live cell migration during extended times postexposure and was thus avoided.

**Figure 3** provides six examples of *in vitro* photothermal damage at 2 µm, each within a narrow range of irradiances (12.15-12.29 W cm^-2^). This laser irradiance range was well within the standard deviations (Section 3.1) and uncertainty for determining 2-µm irradiance (2.85%), and yet there were three positive and three negative damage outcomes. These are referred to as damage crossovers. In all irradiances below and above these two irradiances, cell responses were always negative and positive, respectively (**Table 1**). When crossovers do not occur, or occur in such a narrow range of irradiances, the process is considered deterministic rather than probabilistic. This was a surprising result and might be the consequence of the relatively large beam and long exposure duration.

**Table 1:** Dose response data for 200-s 2-µm exposures.

Irradiance Range (W cm^-2^)	Number of data points	Damage Frequency (%)
1-4	6	0
4-8	18	0
8-12	15	0
12.15-12.29	6	50
12.4-14.0	15	100
14-18	20	100
18-30	9	100
30-50	4	100
Total	93	

The Probit method, which determines probability of generating damage from laser irradiances in future exposures under the same experimental conditions, was unable to determine threshold values for the 2-µm exposures. This was because there were insufficient crossovers in the data set for the algorithm to converge. Therefore, we confined our data to what is summarized in **Table 1**. The mean peak (center) steady-state temperature and temperature rise (34.5 °C ambient) for the three 200-s 2-µm positive damage events in **Figure 3** were 50.0± 0.9 °C and 15.5± 0.9 °C, respectively. The deterministic threshold is the average of the irradiance range (12.22 W cm^-2^). These values established the threshold thermal metrics for damage by purely photothermal mechanisms (2-µm exposures).

Distinguishing features for the photothermal lethal effects shown in **Figure 3** include the contiguous damaged (red) regions in the center of the exposed site (panels A, D, E). All six exposures generated a sublethal “halo” effect at the periphery of the affected areas. At lower irradiances, this halo effect narrows down to a contiguous region of altered morphology and hyperfluorescence. We have chosen to require ethidium (red) staining of cells to indicate overt lethal damage 1-h postexposure. It is important to note there were very few dead cells outside laser exposed areas, as shown in **Figures 2** and **3**.

### 3.2 Determining laser irradiances for concurrent exposures

**Figure 4:**
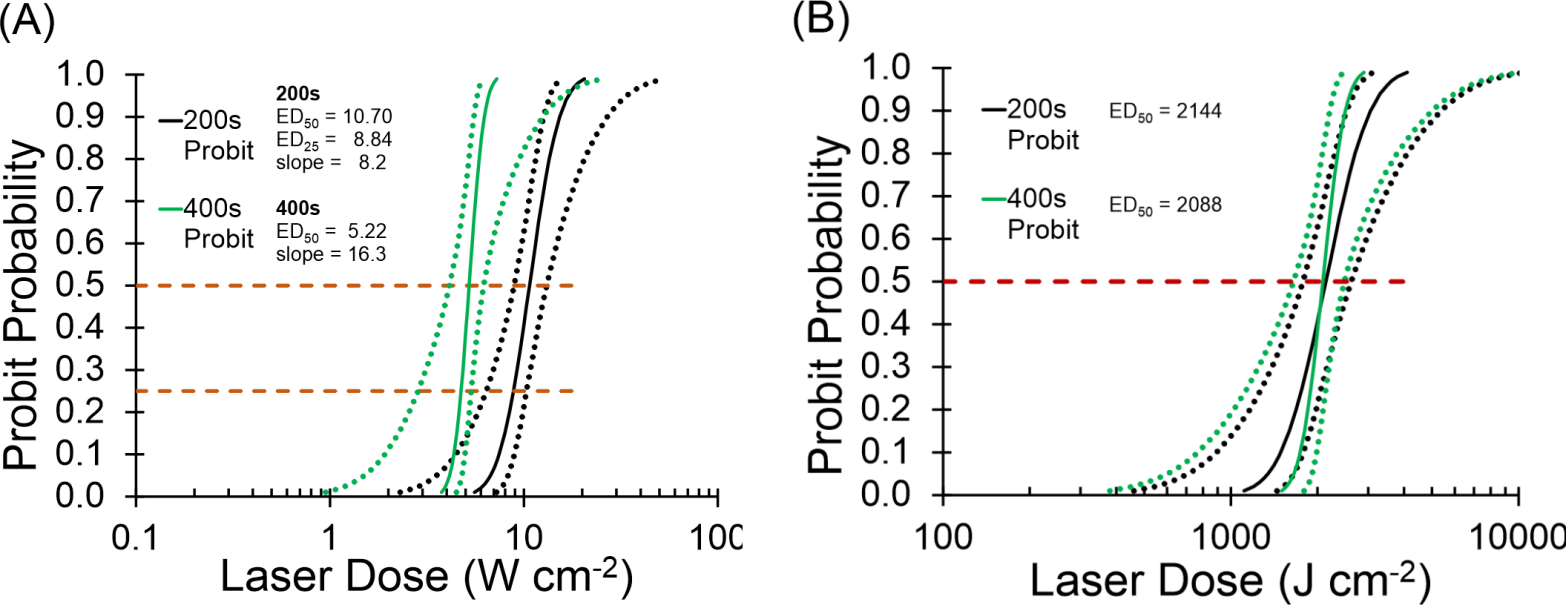
Probit ED curves for 200 s and 400 s at 447 nm. **(A)**: Probit ED_50_ irradiance curves. Black lines are 200 s, green lines are 400 s. Dotted lines represent 95 % confident intervals. **(B)**: Probit ED_50_ radiant exposure curves. Black lines represent 200-s data, green lines represent 400-s data. Dotted lines are 95 % confidence intervals. Dashed lines intersect Probit curves at 25% and 50% probabilities.

The exposure duration for the concurrent exposure experiment was defined by the shortest exposure leading to purely photochemical damage. The purely photothermal exposure at 2 µm would match that exposure duration to generate concurrent laser exposures. **Figure 4** shows the Probit probability curves for 447-nm laser irradiance (**Figure 4A**) and radiant exposure (**Figure 4B**). Irradiance reciprocity was evident between the 200 s and 400 s exposures at 447 nm for the 825-µm diameter beam. In total, 46 and 40 usable 447-nm exposures were tallied at 200 s and 400 s, respectfully. At 200 s, there were 25 positive and 21 negative exposures, ranging from 3.0–100 W/cm^2^. There were 22 positive and 18 negative exposures at 400 s, where irradiance ranged from 1.0–75 W/cm^2^.

The ED_50_ irradiance values for 200 s (10.70 W cm^-2^) was 2-fold greater than the 400-s (5.22 W cm^-2^) value, indicating reciprocity. The overlay of Probit radiant exposure curves in **Figure 4B** further exemplifies reciprocity, where the 200-s value (2144 J cm^-2^) and the 400-s value (2088 J cm^-2^) were nearly identical. The Probit slopes in **Figure 4A** were about 8 (200 s) and 16 (400 s), which identifies sharp probability curves but are only coincidentally different by a factor of two. The relative fiducial limits (FL) for the 200-s and 400-s Probit curves in **Figure 4A** were all less than 30% of their respective ED_50_ values. In addition, the upper FL for the 400-s data was well separated from the lower FL of the 200-s data at the two ED_50_ values (**Figure 4A** upper dashed line). These Probit characteristics indicate that the two Probit ED_50_ irradiances are statistically significant and different from each other with 95% confidence.

Rather than using the threshold ED_50_ irradiance in the concurrent exposure experiment, we wanted to expose with the ED_25_ irradiance. This provided a damage frequency of 25% and therefore allow the range of 25-100% for assessing positive effects of combined exposure with the photothermal beam. This required more damage data (200 s) to be assessed at lower irradiances to bring the lower FL at the probability of 25% to be significant. The data set plotted produced Probit relative FLs within 30% of the ED_25_ value (**Figure 4A** lower dashed line).

To ensure the ED_25_ and ED_50_ values produced damage frequencies at approximately 25% and 50%, respectively, we performed a quick survey. When we delivered 11 exposures at 10.70 W/cm^2^ (200 s ED_50_) and 12 exposures at 8.84 W/cm^2^ (200 s ED_25_) we found 5 positives (46%) and 4 positives (33%), respectively. The ED_50_ damage frequency was at the target, considering we could not score 5.5 positive outcomes to be exactly 50%. A value of 3 positives is needed to be exactly 25% damage frequency. While 4 of 12 does represent one positive response more than predicted by Probit, for our purposes here, the ED_25_ irradiance produced damage about 25% of the time.

As mentioned in the description of **Figure 3**, the peak temperature and temperature rise for the 200-s, 2-µm deterministic threshold at 12.22 W cm^-2^ were 50.0 ± 0.9 °C and 15.5 ± 0.9 °C, respectively. Without Probit ED_25_ and ED_50_ irradiances for 200-s exposures at 2 µm, we had to choose a subthreshold irradiance with meaning for our concurrent exposure analysis. Because the ratio of the 447-nm ED_50_ to ED_25_ irradiances was 1.21, a comparable irradiance for the 2-µm exposure would be 10.1 W cm^-2^. There is no reason to believe that the ratio of ED_50_ to ED_25_ for purely photochemical damage processes would translate directly to the same ratio for purely photothermal processes, so we endeavored to identify an irradiance at 2 µm based on temperature response.

**Figure 5:**
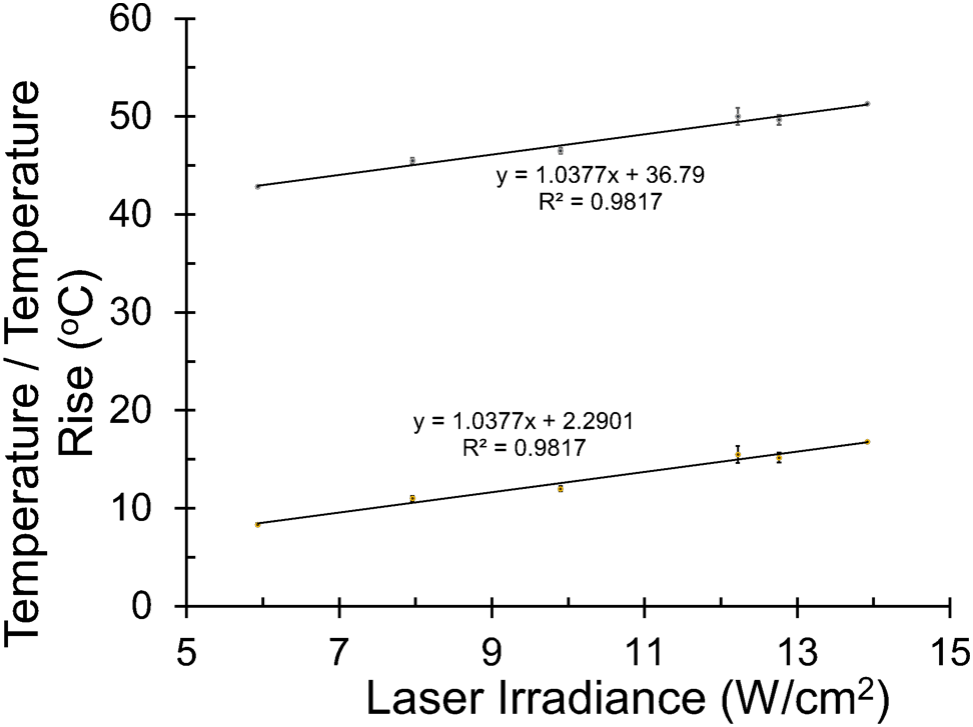
Irradiance-dependent thermal responses at 2 µm. Peak steady-state temperatures were recorded in the center of 2-µm laser exposures at various irradiances. Lines show linearity for temperature and temperature rise (ambient temperature of 35.5 °C).

All exposures of 200 s (477 nm and 2 µm) led to thermal steady-state after about 2 s, and real-time microthermography provided values for the hottest thermal pixel for each exposure. The central steady-state temperatures were termed peak temperatures. **Figure 5** shows the linearity for the 200-s exposures at 2 µm, which provided a standard curve for estimating the irradiance to produce a desired temperature. From **Figure 5**, the temperature expected for a 200-s exposure of 10.1 W cm^-2^ (using the same ratio of the ED_50_/ED_25_ at 447 nm) is 47.3 °C (ΔT=12.8 °C), which is only 5% lower than the threshold 50 °C. This irradiance would likely contribute too much photothermal damage component in the concurrent exposure experiment. Therefore, we chose to use 7.3 W cm^-2^, which should produce a < 10 °C temperature rise (44.5 °C), which is 35% lower than the ΔT threshold and 40% lower than the threshold irradiance for 200 s at 2 µm.

### 3.3 Concurrent exposures

The original design of this experiment was to expose cells to 200-s at the ED_25_ irradiances for both 447-nm and 2-µm beams. Damage frequency and morphology would provide whether the purely photochemical and purely photothermal scenarios were operating fully independently (25% damage), additively (50% damage), synergistically (>50% damage), or in an inhibitory fashion (<25% damage). Unfortunately, damage from 200-s at 2 µm was deterministic (**Table 1**) and an irradiance of 7.3 W cm^-2^ (40% lower than threshold) was chosen in order to generate a temperature rise of < 10 °C (at least 35% lower than threshold), as described in the previous section. Damage frequency and morphology were used to provide insight into damage processes.

In total, 22 simultaneous laser exposures of 8.84 W cm^-2^ at 447 nm and 7. 3 W cm^-2^ at 2 µm were performed. To account for variability, the exposures were evenly spread out over 11 wells of two different 24-well glass bottom plates. The temperatures were monitored for each exposure using microthermography and damage was assessed using viability stain. **Table 2** summarizes the results of the concurrent exposures. Separately, the ED_25_ irradiance at 447 nm produced a damage frequency consistent with the expected 25% rate (4/12 positive) and 7.3 W cm^-2^ 2-μm exposures produced no *in vitro* lesions. Combined, the damage frequency was 100%, indicating synergy between the two damage mechanisms. The average temperatures (and average ΔTs) were 37.3 ± 0.3 °C (2.8 ± 0.3 °C), 44.3 ± 0.7 °C (9.8 ± 0.7 °C), and 46.1 ± 0.6 °C (11.6 ± 0.6 °C), for the 447 nm alone, 2 µm, alone, and the combined exposures, respectively. The highest ΔT for the combined exposures was 12.3 °C. The 1 °C difference between the individual and combined ΔT values was within the error of the thermal camera and is not considered significant.

**Table 2:** Results of concurrent 447-nm and 2-µm 200-s exposures.

τ (s)	λ (nm)	Average Irradiance (W cm^-2^)	Radiant Exposure (J cm^-2^)	Average Peak Temperature (°C)	Average Peak ΔT (°C)	Damage Frequency (positive/total)
200	447	8.84	1768.8	37.3	2.8	4/12
200	2000	7.28	1455.6	44.3^1^	9.8^2^	0/5
200	447	8.84	1768.8	46.1	11.6	22/22^3^
	2000	7.28	1455.6			

Table legend: ^1^Threshold temperature 50.0°C. ^2^Threshold temperature rise (ΔT) 15.5°C. ^3^All damage had purely photochemical morphology.

In addition to the remarkable increase in damage frequency, the morphological features resulting from the combined exposures also provided information regarding cellular processes leading to damage. Some damage indicated maximal photochemical morphology with no intermixed live cells, while others had evenly mixed live and dead cells. All 22 damaged regions were about the same diameter as the 447-nm beam, where 7 lesions had 10-70% cell lethality (red versus green stain) and 15 lesions showed ≥ 95% dead cells.

**Figure 6:**
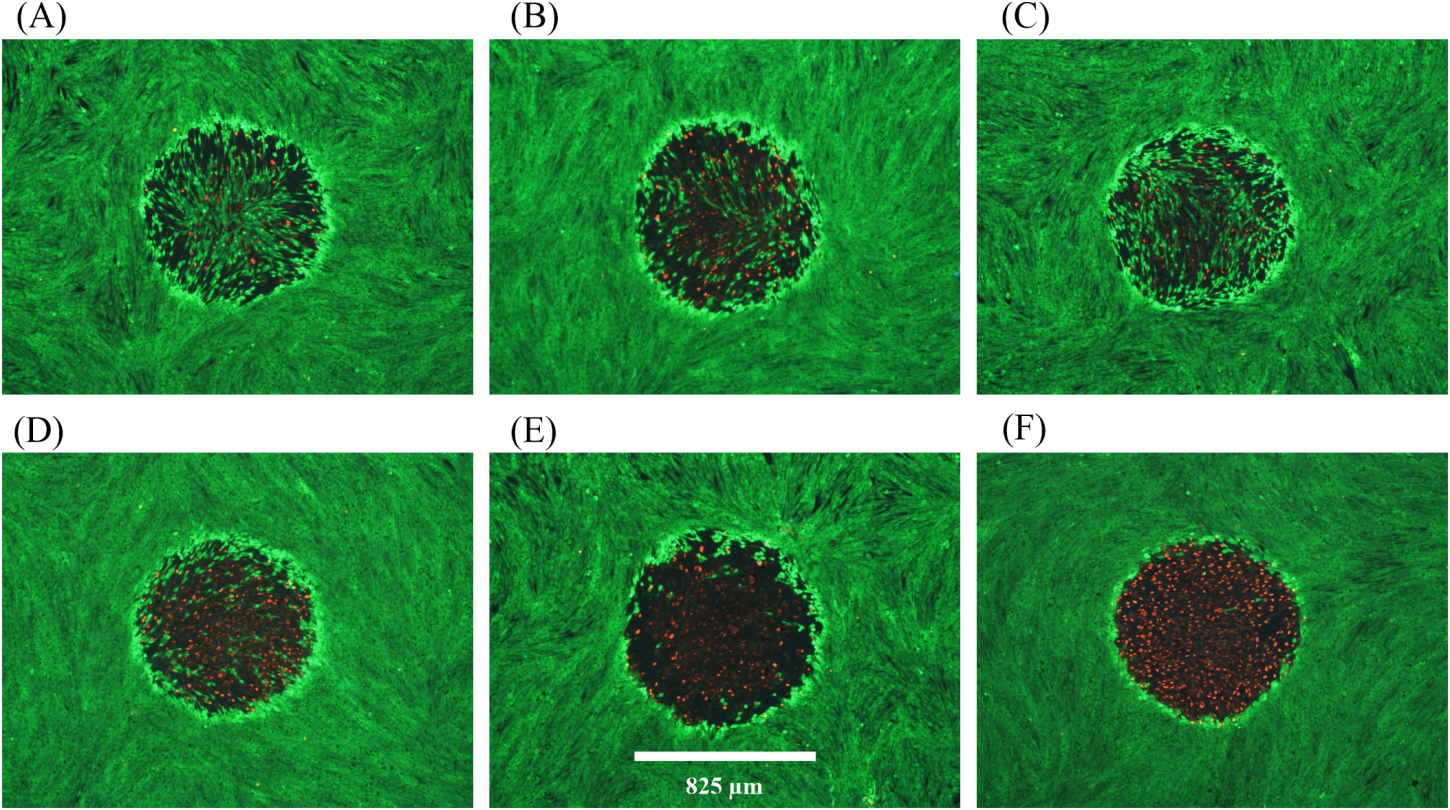
Damage images from concurrent 200-s exposures at 447 nm and 2 µm. A total of 22 wells were exposed to combined laser light at 8.84 W cm^-2^447 nm (Probit ED_25_) and 7.3 W cm^-2^ 2 µm. All 22 wells had damage. **(A–F)** show examples of resulting damage morphology. Spatial bar: 825 µm (beam diameter).

**Figure 6** shows fluorescence damage images for six of the 22 damage events resulting from combined exposures. The images represent a broad range of what was previously described (**Figure 2**) as photochemical in nature. Specifically, the least severe damage (**Figures 6A, C**) was still dramatically greater than the damage commonly seen with 8.84 W cm^-2^ of 447 nm alone (**Figure 2B**). Some of the damaged outcomes (**Figures 6B, D**) exhibited similar morphology to those from the 447 nm ED_50_ exposures (**Figure 2C**). **Figure 6E** represents damage seen previously only at irradiances 50-100% above the 447-nm ED_50_. Finally, most (15/22) damage outcomes from combined exposures had lesions with >95% cell lethality within the exposed region (see **Figure 6F**).

The results of **Figure 6** suggest that, although there was a significant temperature rise associated with the concurrent exposures, photochemical mechanisms predominated for causing cell death. Due to the lack of *in vitro* photothermal damage morphology we cannot comment on a contribution to cell death by thermal mechanisms. The temperature rises achieved by the combined exposures were about 4 °C below the established photothermal threshold of 15.5 ± 0.9 °C, which also suggests a damage mechanism of predominantly photochemical nature. Although this is a distinct example of photochemical damage enhanced by elevated temperature, it remains unclear if subthreshold photothermal processes were taking place in parallel with the photochemistry.

Overall, the results of the concurrent laser exposures undoubtedly argue against the notion that photochemical processes are confined to nonthermal mechanisms. Choosing extended laser exposure durations at the two wavelengths, each representing pure damage processes, permitted a deductive analysis of the damage results. Even though the intracellular MPs were not required for the bulk water heating at 2 µm, the melanin granules may play a role in damage at the elevated temperatures. Our results do not distinguish whether the cause of damage was from enhanced photochemistry at redox enzymes (6) or direct oxidation reactions on proteins (7). Likewise, elevated temperature may have accelerated photochemical reactions at the melanosomes (1, 8–10). Although additional work is needed, including concurrent exposures in nonpigmented cells, our results indicate a high probability for thermal acceleration of photochemical processes when pigmented cells are exposed to lasers emitting light in the blue spectrum.

### 3.4 Threshold temperatures

The premise of this experiment is that, when laser photons have sufficient energy to damage cells by either photothermal or photochemical (i.e. blue light) mechanisms, the extent of the thermal requirement can be reduced when photochemical mechanisms contribute to damage overall. In the progression from purely photothermal (highest temperature) to purely photochemical damage (least requirement on temperature), the intermediate scenario of mixed damage mechanisms would have an intermediate temperature rise. We have shown that progression from short to long exposure durations at 413 nm revealed a transition in damage mechanisms, with a sharp transition to purely photochemical between 60–100 s (17, 19). Simulated temperatures using Probit ED_50_ irradiances showed a transition from very high (>24°C for 0.1-1.0 s) to intermediate (17-13 °C for 20–60 s) prior to the sharp transition (<3.5 °C for >100 s) (19). Unfortunately, there was no photothermal control. Here, we assessed thermal responses of cells exposed at 447 nm and 647 nm for 1, 60, and 100 s, where the 647-nm data would represent a purely photothermal standard for heating via MP absorption. Additionally, we determined threshold temperatures, indicating the minimal thermal requirement for damage overall.

The centrally located contiguous region of red fluorescence seen after 2-µm exposures in our pigmented RPE cell monolayer (**Figures 3A, D, E**) is a distinguishing feature for photothermal damage. Temperature rises are greatest in the center of an exposed site, with a gradient of lower temperatures expanding radially. Previously (23, 24), we have described how to determine the threshold temperature associated with the boundary of cell death, where contiguous dead (red) and live (green) cells converge in postexposure fluorescence images. The threshold temperatures were found to be dependent upon laser exposure duration and to some extent the damage mechanism, such as pigment absorption (VIS wavelengths) versus bulk water heating (e.g. 2 µm). The thermal requirement for death from exposure to 447 nm was compared to that for exposure to 647 nm. A reduced thermal requirement suggests contribution from photochemical processes and thus, indicating combined damage processes.

**Figure 7:**
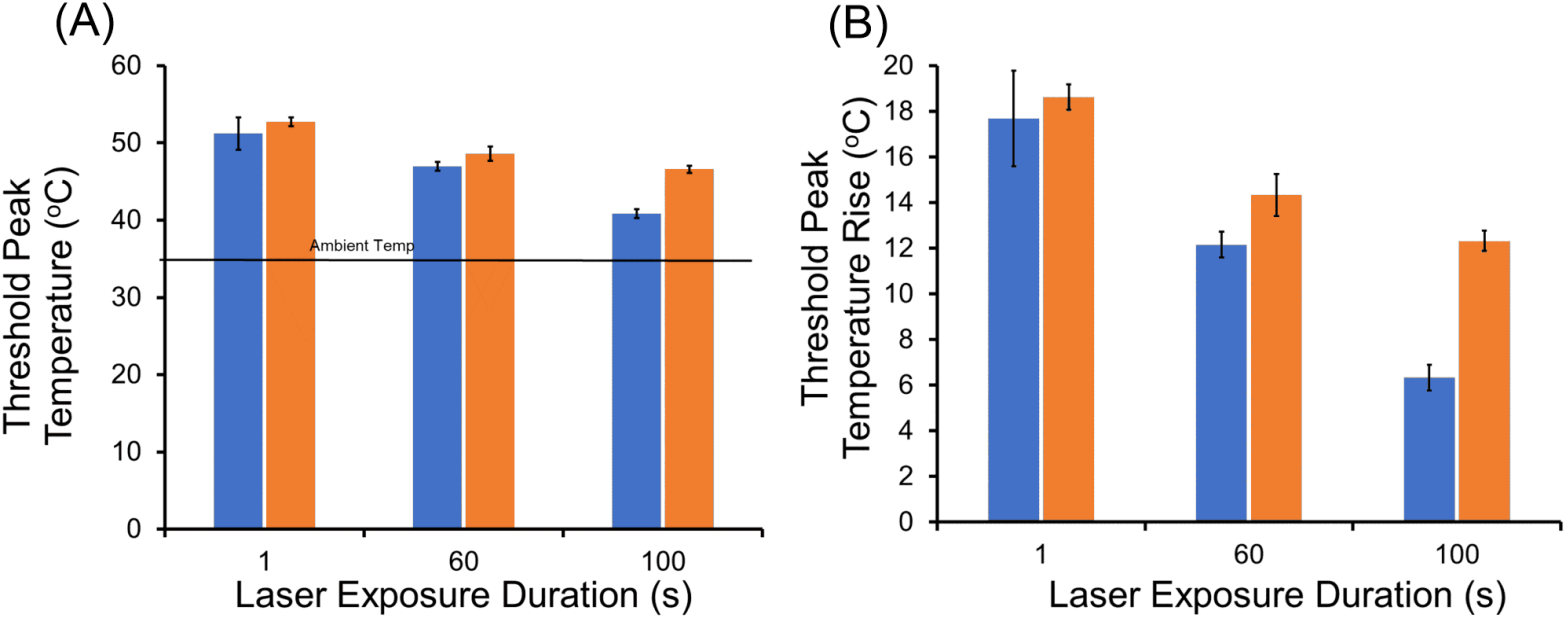
Threshold peak temperatures (boundary of cell death) at 447 nm and 647 nm. **(A)**: Threshold peak temperatures for 1, 60, and 100-s exposures; blue bars are 447 nm and orange bars are 647 nm. Ambient temperature was 35.5 °C. **(B)**: Threshold peak temperatures rise for 1, 60, 100-s exposures; blue bars are 447 nm and orange bars are 647 nm.

Threshold temperatures at the end of laser exposures (threshold peak temperatures) at 447 nm and 647 nm are shown in **Figure 7**. It is important to remember that none of the 447-nm exposures at 1–100 s were purely photochemical due to a lack of irradiance reciprocity. With limited laser powers, we used smaller beam diameters for this experiment (447 nm: 416 ± 8 µm, 647 nm: 415 ± 21 µm) as described in Section 3.1. Identical beam diameters eliminated differences in heat dissipation rate seen between different diameters, known as the spot size effect.

As expected for the photothermal control (647 nm), threshold temperature was reduced as exposure duration was extended. This follows the well-established dogma that thermal damage depends on a combination of both temperature and time (temperature history). Both threshold peak temperature (**Figure 7A**) and threshold peak ΔT (**Figure 7B**) are shown. For the 1-s exposures, there was no statistical difference between the threshold peak temperatures for the two wavelengths, indicating both generated purely photothermal damage. Starting with 60-s exposures, a measurable time-dependent reduction in threshold peak temperature and ΔT was found for the exposures at 447 nm relative to 647 nm. This reduction revealed a decreased requirement for thermal damage contribution for the blue wavelength, which at 100-s was reduced to half of that required at 647 nm. Specifically, the statistically significant (p=0.002) difference in threshold peak ΔT at 60 s was 2.0 °C (14.3 ± 1.0 °C versus 12.3 ± 0.9 °C). For exposures of 100 s, the statistically significant (p<0.0001) difference in threshold peak ΔT was 6.0 °C (12. 3 ± 0.7 °C versus 6.3 ± 0.6 °C).

The results from the threshold peak temperature analysis revealed a significant reduction in thermal contribution required to induce damage at 447 nm. This moderation in thermal requirement for overall cellular damage suggests a compensatory increase in a different damage mechanism. Clearly, photons at 447 nm have sufficient energy to produce photochemical damage, so it is likely that this compensation involves increased oxidative stresses. Again, our qualitative analysis cannot provide insight into the exact mechanism for the 447-nm damage compensation at intermediate exposure durations (e.g. RXS production, direct protein oxidation, photosensitization via MPs), but our data suggest a blend of the two mechanisms.

### 3.5 Conclusions

We have investigated the combined photothermal and photochemical damage mechanisms for pigmented RPE cells exposed to blue laser light using an *in vitro* model of laser eye damage. The model does not account for the variety of features of RPE cells in the eye and was used because of its simplicity to study laser damage mechanisms. Similarly, the extended duration of laser exposures used in this study was not indicative of real-world ocular exposures but was useful in producing purely photochemical damage processes for analysis.

We found greatly enhanced photochemical damage at elevated temperatures (46 °C) and a reduction in the thermal damage component for intermediate exposures (60 s and 100 s) at 447 nm. Both results were compared to wavelengths expected to produce purely photothermal damage as controls. Unexpectedly, the use of large diameter laser beams provided key *in vitro* morphological features that distinguished between photothermal and photochemical damage. Also surprising was that for long exposures (200 s), damage from bulk water heating at 2 µm was deterministic, and the Probit method for probabilistic ED_50_ determination was inappropriate.

Our *in vitro* results distinctly show that photochemical damage processes are not confined to nonthermal states, but do not provide mechanistic clues regarding how elevated temperature accelerates that damage. Unfortunately, our results do not provide insight into whether distinct damage pathways exist for photothermal and photochemical damage. Rather, they imply the photochemical pathway to death is enhanced by elevated temperature, and that the mechanistic processes involved are not inhibited or damaged by relatively high temperature (46 °C). Our results support the assertion from Ham’s group in 1979 that combined damage mechanisms are likely for intermediate temperature rises (10-20°C) (13).

Finally, additional studies are needed to support possible changes in national and international laser safety standards to reflect the growing body of evidence that thermal and photochemical damage mechanisms coexist in cells. Future experiments will focus on *in vitro* methods to identify concurrent damage processes in shorter laser exposure durations that more accurately mimic possible unsafe ocular exposures.

## Data Availability

The original contributions presented in the study are included in the article/supplementary material. Further inquiries can be directed to the corresponding author.
